# Supplementary material for: Are carbon emissions trading and green financial instruments synergistic? -Comprehensive quantitative research based on content analysis
Source: PLoS One. 2024 Mar 7;19(3):e0298601. doi: 10.1371/journal.pone.0298601 (PMC10919601; doi:10.1371/journal.pone.0298601)
Supplement: S1 Data — (ZIP) [file pone.0298601.s001.zip › Data/地级市Z_F工作报告.zip!/╜¡╬≈/╥╦┤║╩╨/└┤╫╘╣1⁄2╓┌║┼í╛┬φ┐╦╩2╛▌═°í┐_2017.docx]

各位代表：

　　现在，我代表市人民政府，向大会报告工作，请予审议，并请各位政协委员和列席会议的同志提出意见。

　　一、2016年工作回顾

　　过去的一年，在省委、省政府和市委的坚强领导下，全市上下认真贯彻习近平总书记系列重要讲话精神和治国理政新理念新思想新战略，特别是对江西工作提出的“新的希望、三个着力、四个坚持”重要要求，紧紧围绕建设美丽宜春决胜全面小康奋斗目标，积极谋发展、比实干、争先进，统筹做好稳增长、调结构、促改革、优生态、惠民生等各项工作，经济社会保持平稳健康发展，实现了“十三五”良好开局。

　　经济运行稳中有进。全市生产总值1770.4亿元，增长9%。财政总收入320.9亿元，同口径增长10.1%，其中一般公共预算收入220亿元，同口径增长10.9%。500万元以上固定资产投资1821.4亿元，增长14.7%。社会消费品零售总额596.3亿元，增长11.8%。财政总收入超20亿元县（市、区）达到7个。丰城位居全国县域经济与县域基本竞争力第75位，樟树跻身全国县域经济与县域基本竞争力百强县, 高安列为全国县域经济投资潜力百强县。

　　产业结构逐步优化。三次产业比由上年的14.6:51.7:33.7调整为14:50.9:35.1。全市农业总产值440.6亿元。规模以上工业增加值956.2亿元，增长9.2%，其中高新技术产业增加值达到240亿元，增长9.5%。服务业增加值占生产总值比重提高1.4个百分点。

　　城乡建设扩容提质。预计全市城镇化率达到46.5%，提高1.7个百分点。中心城区常住人口达到57.4万人。丰城、樟树获评中小城市新型城镇化质量百强县，温汤、华林山、宝峰分别入选全国首批特色小镇、全国美丽宜居小镇和全国特色旅游景观名镇。

　　人民生活持续改善。城镇新增就业5.8万人，登记失业率控制在3%以内。居民消费价格总水平涨幅2.5%，控制在调控目标内。城镇、农村居民人均可支配收入分别达到27452元和12643元，增长8.2%和8.8%。

　　一年来，主要做了以下工作：

　　（一）壮大经济总量，产业升级步伐加快。坚持不懈推进“三升级”，产业层次和水平不断提高。工业经济量质齐升。新增“专精特新”中小企业112户、规模以上工业企业221户，分别位居全省第一和第二。预计工业园区实现主营业务收入3600亿元，奉新工业园区列入长江经济带国家级转型升级示范开发区，高安建陶产业基地获评国家新型工业化产业示范基地。七大支柱产业主营业务收入达到3000亿元，锂电新能源、中医药产业分别达到150亿元和280亿元。现代农业优势彰显。粮食总产达到85.8亿斤，粮食托市收购量全省第一。肉牛出栏46万头，绿色食品原料基地、有机农产品认证、富硒农产品基地、高产油茶林面积分别达到138万亩、95万亩、21万亩、88万亩。新增“三品一标”认证数68个，居全省第一。累计发放“财政惠农信贷通”贷款31.4亿元，获评全省先进设区市。市级以上农业产业化龙头企业突破350家，注册农民合作社6450家，家庭农场达到1741家。万载评为全国农村产业融合发展试点示范县。146座小型水库除险加固项目全部完工。农村土地确权颁证率达到98%。获评全省耕地保护成效显著设区市，靖安、高安、宜丰荣获全国国土资源节约集约模范县。旅游服务业日趋活跃。成功举办第10届月亮文化旅游节，全年接待游客5298.7万人次，实现旅游综合收入431.4亿元，分别增长37%和43.4%。明月山入选全国十大最美赏月地，靖安、铜鼓、宜丰列入国家全域旅游示范区创建名单，袁州获评全国休闲农业与乡村旅游示范县，万载恒晖大农业评为全国休闲农业与乡村旅游五星级企业。电子商务交易额突破300亿元、增长50%，铜鼓获评全国电子商务进农村示范县。新增A级物流企业7家，总数达到24家。

　　（二）提升人居环境，城乡面貌有效改观。坚持以城带乡、城乡统筹，深入推进新型城镇化建设。城市功能日益完善。铜万高速、东昌高速、320国道绕城改建竣工通车，220国道绕城改建顺利开工，四方井水利枢纽工程、龙头山水电站枢纽工程、明月山机场站坪扩建、黄颇路棚户区改造有序推进，万达城市综合体项目开业运营。中心城区新建人行天桥3座、地下通道1个，路网通行能力不断提升。公共自行车租赁系统建成并投入使用。城市治理得到加强。中心城区卫生环境有效改善，顺利通过国家卫生城市复审。启动城市建成区违法建设专项治理工作，拆除违法建设面积8.8万平方米，非法三轮车载客营运得到有效治理。全面开展老楼危楼排查整治，宜春商城农贸市场平稳关停。全市商品住宅库存平均去化时间比2015年底减少10个月。城乡环境不断优化。县道升级改造全面完成，新建25户以上自然村通水泥路1200公里，改造农村公路危桥128座。顺利完成省定63个中心村和558个一般自然村点建设任务。全面推行“河长制”，大力实施“清河行动”，袁河、肖江、潦河等重点流域环境综合治理成效明显。靖安、铜鼓纳入国家重点生态功能区并获批国家级生态县，宜丰通过国家级生态县验收。

　　（三）推进改革开放，发展活力竞相迸发。坚持以改革破解难题，以开放促进发展，以创新激发活力。各项改革蹄疾步稳。市本级行政权力事项、行政审批事项分别精简60.9%和71.1%。事业单位分类改革、公务用车改革、国资国企改革全面推进。启动实施“五证合一、一照一码”登记制度改革，新增市场主体36477户、注册资本556.9亿元。退出粗钢产能18万吨，关闭煤矿65处、退出产能359万吨，完成省政府下达的目标任务。扎实开展降成本优环境专项行动，累计为企业减负54亿元。市级农村综合产权交易中心建成启用。36家公立医院全部取消药品加成。开放合作力度加大。深入实施招商引资“百日攻坚”，组织参加绿发会、药交会、赣港会、赣台会等大型招商活动，实际利用外资达到7.1亿美元，引进省外2000万元以上项目269个、资金569亿元。外贸出口突破155亿元。宜春海关开关运营前期工作稳步推进，江西（宜春）—福州（江阴港）快速班列顺利开通。成功举办了湘赣边区域开放合作联席会。创新创业加速推进。新增国家高新技术企业75家，新组建市级科技创新平台和创新团队37家，获批省级工程技术研究中心5家和重点实验室1家。发放创业担保贷款17.4亿元，直接扶持1.2万人创业，带动5.5万人就业。金融机构贷款余额达到1566亿元，增长18.8%。新增13家企业在“新三板”挂牌，居全省第二。

　　 （四）增进民生福祉，社会大局和谐稳定。坚持新增财力向民生倾斜，着力保障改善民生。社会保障更加有力。十类民生项目100件实事基本完成。城乡居民基本养老保险参保率达到99.1%，零就业家庭就业安置率继续保持100%。完成扶贫移民搬迁6008人，全年减贫5.2万人。新开工棚户区改造16236套，基本建成保障性住房20359套。社会事业显著进步。新改扩建中小学72所、新增学位3.2万个，9个县（市）通过全国义务教育发展基本均衡县认定，达标率居全省第一。率先在全省完成直播卫星“户户通”工程，宜春采茶戏《南田印象》获得国家艺术基金资助。入选“中国好人榜”153人。成功举办全国农耕健身大赛和市第五届全民健身运动会，获批国家健康城市首批试点城市。樟树、宜丰荣获全国计划生育优质服务先进单位。万载获评全国双拥模范县。综合治理取得实效。安全生产保持总体平稳态势。地质灾害防治连续八年实现“零死亡”。深化“平安创建”活动，重拳打击“盗抢骗”，公众安全感得到提升。创新信访工作体制机制，各类矛盾纠纷化解率达到99%，化解信访积案“1+1+N”工作模式在全国推广。

　　 （五）加强自身建设，行政效能得到提升。坚持转变作风，优化政务服务，着力建设人民满意政府。政府职能加快转变。深入开展“两学一做”学习教育，干部作风明显好转。加快推进“双随机、一公开”，行政监管更加规范。积极推动审批服务事项进驻行政服务中心集中办理，服务效率进一步提高。狠抓政府执行力建设，严格督导追责问效。依法行政深入推进。健全完善市政府有关工作制度，政府工作规范化、制度化、科学化水平明显提升。全面推进法治宜春建设，法律顾问制度实现市县乡村四级全覆盖。认真办结人大代表建议、政协委员提案，满意和基本满意率达到100%。廉政建设扎实有效。严格落实中央八项规定、国务院“约法三章”和省委有关规定，“三公”经费进一步下降。认真履行“一岗双责”，强化行政监察和审计监督，政府廉政建设和反腐败工作取得新成效。

　　与此同时，国防动员和后备力量建设、人民防空、拥军优属、国家安全、民族宗教、外事侨务等工作持续加强；邮政通信、电力、石油、烟草、气象、水文、盐业、无线电管理、防震减灾、统计、档案、史志、老龄、残疾人和红十字会等工作取得新进展。

　　奋斗历尽艰辛，发展凝聚智慧。一年来，我们取得的各项成绩，源自于省委、省政府和市委的正确领导、科学决策，得益于市人大、市政协和社会各界的有效监督、鼎力支持，归功于全市广大干部群众的团结一心、奋力拼搏。在此，我代表市人民政府，向全市人民，向各位人大代表、政协委员和离退休老同志，向各民主党派、工商联、人民团体，向驻宜部队官兵、公安干警，向所有关心、支持宜春发展的各界人士，表示衷心的感谢并致以崇高的敬意！

　　在总结成绩的同时，我们要清醒地看到，经济社会发展还存在不少的困难和问题，主要是：经济总量依然偏小，发展不足的矛盾较为突出，特别是市本级经济实力还比较弱；产业转型升级任重道远，缺乏具有明显支撑作用的优势产业，至今没有过百亿的龙头企业；改革创新仍需攻坚，一些体制机制障碍尚未破除，创新驱动和内生增长动力仍显不足；城乡基础设施还不够完善，城市建设管理水平有待提升；保障改善民生的压力不小，脱贫攻坚任务比较繁重；政府部门少数干部主动服务和担当意识不强、工作落实不力，行政效能有待进一步提高；安全环保形势严峻，特别是去年底连续发生的袁州北槽煤矿“11·22”透水事件和上高自来水异常事件，让我们深感痛惜和自责。对于这些困难和问题，我们要高度重视，动脑筋、想办法，切实予以解决，使政府工作更加符合发展需要和人民期盼。

　　 二、2017年主要工作

　　2017年是实施“十三五”规划的重要一年，是推进供给侧结构性改革的深化之年，是本届政府全面履新的开局之年，做好今年各项工作意义重大。从国内来看，经济发展呈现速度换挡、结构优化、动力转换三大特征，尽管外部经济环境错综复杂、下行压力依然较大，但我国经济发展长期向好的基本面没有改变。从全省来看，省第十四次党代会顺利召开，正引领着全省上下同心同德、砥砺前行，奋力迈出决胜全面建成小康社会、建设富裕美丽幸福江西的坚实步伐。从自身来看，市第四次党代会提出了坚持“五个一”、建成“五个区”，实现主要经济指标“提前翻番、力争百强”的奋斗目标，描绘了建设美丽宜春决胜全面小康的宏伟蓝图，为做好政府工作指明了方向、明晰了路径。我们要牢牢抓住发展的“窗口期”，以时不我待的紧迫感和只争朝夕的精气神，找准“新坐标”，奋力推动赶超发展；培育“新优势”，加速集聚发展动能；下好“先手棋”，加快释放改革红利；高举“生态牌”，彰显绿色发展优势；打好“主动仗”，提升安全环保水平；织密“保障网”，促进民生普惠共享，不断开拓宜春发展新境界。

　　今年政府工作的总体要求是：以党的十八大和十八届三中、四中、五中、六中全会精神为指导，深入贯彻习近平总书记系列重要讲话精神和治国理政新理念新思想新战略，特别是对江西工作的重要要求，认真落实中央经济工作会议、全省经济工作会议部署和“四个始终”要求，坚持稳中求进工作总基调，牢固树立和贯彻落实新发展理念，主动适应经济发展新常态，坚持以深化供给侧结构性改革为主线，积极推进“三升级、三高于、两加强、一同步”和“三区两核一盘棋”举措，统筹稳增长、促改革、调结构、优生态、惠民生、保安全各项工作，推动经济平稳健康发展和社会和谐稳定，奋力迈出建设美丽宜春决胜全面小康的坚实步伐，以优异成绩迎接党的十九大胜利召开。

　　今年全市经济社会发展的主要预期目标是：生产总值增长9%以上；财政总收入增长6%，其中一般公共预算收入同口径增长5%左右；规模以上工业增加值增长9.5%左右；500万元以上固定资产投资增长15%左右；社会消费品零售总额增长12%；实际利用外资增长9%以上；外贸出口增长1%以上；城镇居民人均可支配收入增长8.8%左右；农村居民人均可支配收入增长9.5%左右；居民消费价格总水平涨幅控制在3%左右；城镇登记失业率控制在4%以内；节能减排完成省下达的计划任务。

　　实现上述目标和要求，重点要抓好以下八个方面：

　　（一）主攻转型升级，推动产业提质增效

　　推动产业转型升级是宜春经济发展面临的重大而紧迫的任务。要紧紧围绕做大增量、做强存量、做优品质，深入实施工业强市战略，加速培育现代农业和旅游服务业发展新优势。

　　大力发展新型工业。实施强攻工业制造升级三年行动，大力发展新制造经济，加速推进新型工业化。力争规模以上工业增加值、主营业务收入、利税总额分别达到1040亿元、4300亿元和540亿元。举全市之力打造“亚洲锂都”，尽快出台《关于加快推进锂电新能源产业发展的实施意见》，设立锂电新能源产业发展引导基金，定期召开锂电新能源产业发展调度会，把锂电新能源产业作为强攻工业的首位产业来推进，重点在资源管控利用、龙头企业培育、产业链招商、高端人才引进、关键技术攻关上下功夫、求突破，力争主营业务收入达到260亿元。全面振兴“中国药都”，设立中医药产业发展引导基金，把中医药产业作为强攻工业的支柱产业来打造，加强中药资源保护、研究开发和合理利用，推动中药材种植绿色发展和中医药产业转型升级，力争主营业务收入达到330亿元。注重把绿色食品产业作为强攻工业的特色产业来培育，以建设绿色食品产业基地为抓手，大力推行绿色种植、生态养殖，积极发展精深加工，不断壮大绿色食品产业集群。利用云计算、物联网、大数据等新技术，改造提升机电、建材、化工、纺织服装等传统优势产业，支持工埠机械、华伍股份等企业加快发展，力争七大主导产业主营业务收入占全部工业的比重提高2个百分点。稳步实施工业园区提升计划，完善功能配套，促进集约发展，力争主营业务收入达到4000亿元。积极开展挂园联企帮扶活动，培优扶强骨干企业，力争新增规模以上工业企业150户以上，主营业务收入超10亿元企业达到90户，争取实现百亿企业零的突破。

　　做优做特现代农业。围绕将宜春打造成全省现代农业先行区，加快构建现代农业产业体系、生产体系和经营体系，突出品牌创建，做足特色文章，真正实现“农业亮市”。力争全市农业总产值达到463亿元，粮食总产稳定在85亿斤以上。深入实施藏粮于地、藏粮于技战略，巩固全国粮食主产区地位。加快发展优势特色产业，力争肉牛出栏达到60万头，绿色食品原料基地、有机农产品认证、富硒农产品基地、高产油茶林、中药材种植面积分别达到190万亩、100万亩、25万亩、118万亩、30万亩。实施品牌振兴、龙头引领行动，聚力打造“宜春油茶”、“宜春大米”、“宜春中药材”等原产地地理标志保护品牌。稳步实施绿色生态农业“十大行动”，着力强化农产品质量安全监管，争创国家农产品质量安全市（县）。培育壮大农业龙头企业，改造提升现代农业示范园区，推动一二三产业融合发展。扶持家庭农场等新型农业经营主体发展，提高农业现代化设施装备水平，促进农业适度规模经营。

　　加快提振旅游服务业。坚持走旅游服务业兴市之路，打造后明月山时代，构筑旅游服务业发展新高地。力争全年接待游客7300万人次，实现旅游综合收入603亿元，服务业增加值达到710亿元。抓紧启动全市游客集散中心和智慧旅游平台建设，着力完善旅游服务功能，努力将宜春打造成国际知名健康养生休闲度假旅游目的地。创新办节模式，发挥社会资源优势，提升月亮文化品牌效应。深入挖掘禅宗文化内涵，加快推进明月山洪江禅意特色小镇建设，进一步叫响“禅宗圣地”旅游品牌。扎实推进景区改造提升，合理开发和利用明月山富硒温泉水，争取温汤镇成功创新中国成立家级旅游度假区、国家温泉旅游名镇、世界温泉健康名镇。推动靖安、铜鼓、宜丰创新中国成立家全域旅游示范区，支持靖安三爪仑争创5A级旅游景区，力争全市新增4A级旅游景区3—4个。大力培育新服务经济、分享经济和智慧经济，促进现代物流、电子商务、家政服务等融合发展，支持袁州、宜春经开区打造高铁与临空经济融合发展示范区。深入实施县域金融“六个一”工程，力争引进外埠银行1家、设立村镇银行2家以上，新增境内外主板上市企业1家、“新三板”挂牌企业10家以上。

　　（二）扩大开放合作，增强投资拉动作用

　　扩大开放是实现赶超发展的必由之路。要坚持“引进来”与“走出去”协调互动，充分发挥投资的关键作用，切实增强赶超发展后劲。

　　加强区域合作。主动对接融入“一带一路”、长江经济带等国家战略，进一步拓展发展空间。扎实推进赣西经济转型加快发展合作项目，推动赣西区域一体化发展。促进宜春海关早日开关，加快国家级烟花爆竹检测重点实验室、鞋类检测区域中心实验室和有机食品农产品区域实验室的建设步伐，推进万载公路口岸、高安铁路口岸作业区建设。进一步密切与九江、莆田等地的交流合作，尽快打通物流新通道。鼓励和支持有条件的企业“走出去”，不断开拓“一带一路”沿线国家市场。

　　突出招大引强。围绕产业升级需求，实施招商引资重大项目集中攻坚行动，重点瞄准世界500强、国内500强和民营企业500强及科技水平含量高的企业，有针对性地开展招商引资，尽快促成正威国际等项目落地建设。精心策划系列专题招商活动，充分利用药交会、赣港会、赣台会、首届世界赣商大会等省级招商平台，着力引进一批重大项目。积极推进“央企入宜”，加快中节能、中汽零、中电投等项目建设步伐。深入实施宜商回归工程，鼓励支持异地商会建设，广泛动员在外宜春籍客商回乡创业。健全完善招商引资奖励机制，调动社会各界参与招商引资、服务项目建设的积极性。

　　拓宽融资渠道。既要发挥好政府投资的带动作用，更要拓展多元化的市场融资渠道，有效解决投入不足的问题。鼓励和引导各县（市、区）在风险可控的前提下，设立和引进股权投资基金、产业发展基金和风险投资基金。在充分利用好商业性金融的同时，积极争取政策性金融和开发性金融支持，加快推进“险资入宜”。大力推广应用PPP模式，最大限度撬动社会资本。

　　优化投资环境。推进行政许可标准化建设，畅通重大项目审批绿色通道，坚决整治中梗阻、不作为、慢作为等现象。大力推进“互联网+”政务服务，稳步实施“五证合一、一照一码”登记制度改革，最大限度便企利民。完善重大项目协调推进机制，强化项目跟踪服务，及时协调解决项目建设中遇到的困难和问题。

　　（三）统筹城乡发展，构建宜居宜业家园

　　促进城乡协调发展是建设美丽宜春的核心要义。要突出规划引领作用，加快推进以人为核心的新型城镇化，努力构建城乡一体化发展新格局。

　　提升城市建设水平。按照“统筹布局、规划先行、宜万同城、板块设计、彰显文化、提升品位”的要求，做优做美中心城区，打造宜居宜业宜游区域性中心城市。扎实推进“多规合一”，抓紧启动中心城区总体规划修编和宜万同城发展战略规划编制，积极做好宜万快速通道建设前期工作。加快推进城市“双修”工作，全面开展提升城市功能三年行动，切实增强中心城区的承载力和吸引力。遵循“只拆不建”的原则，加快老城区改造提升，进一步增强服务功能。加大棚户区改造力度，突出抓好灵泉池历史文化街区等棚户区改造项目，以货币化安置为主，积极探索“房票”模式，努力消化房地产库存。深入开展老旧危楼排查整治，分期分批拆除空心预制板危房建筑。优化城区路网结构，优先发展公共交通。推动城市建设与人民防空融合发展，启动市本级人防疏散基地建设。

　　促进城乡一体发展。大力支持上高“撤县设市”。推动城乡基础设施同步设计建设、一体运营，提高“水电路气通信”等城乡联网和通达水平。加快完善乡镇公共服务体系，突出抓好全国重点镇、全省百强中心镇建设，打造一批融历史、人文、产业、生产、生活、生态为一体的特色小镇。按照“整洁美丽、和谐宜居”的要求，扎实推进新农村建设。加快集镇污水处理设施建设，持续改善农村人居环境。从严加强农村住房建设管理，有效规范农民建房秩序，全面提升乡村建设水平。

　　完善基础设施体系。围绕打造区域性综合交通枢纽，加快推进220国道绕城改建、明月山机场站坪扩建、蒙华铁路煤运通道岳吉段和昌吉赣客专建设，抓紧启动靖安至樟树加密高速前期工作。强化能源保障支撑，重点推进110千伏城中变电站、龙头山水电站、太阳岭风电场、靖安洪屏抽水蓄能电站二期和奉新抽水蓄能电站等项目建设。完善水利设施和农村道路，加快四方井水利枢纽、五河防洪、小型病险水库除险加固等项目建设，实施农村公路通达工程。

　　理顺城市治理体制。加快城市执法体制改革，实现职能下放、重心下移，构建管理更加顺畅、责权利更加明晰的体制机制。稳步推进环城东路地下综合管廊建设，争取列入全省海绵城市建设试点。加强智慧城市建设，形成综合性城市管理数据库。以城乡结合部为重点，从治理脏乱差入手，大力实施美化亮化净化工程，推动城市环境面貌持续改善。深入推进城市建成区违法建设专项整治行动，坚决遏制新增违法建设，确保2017年底查处不低于违法建设总量的60%。

　　（四）推进改革攻坚，激发经济社会活力

　　深化改革是加快经济转型升级的关键。要增强改革定力，保持改革韧劲，确保各项改革任务落地见效。

　　着力推进关键性改革。突出抓好供给侧结构性改革，全面落实“三去一降一补”任务。重点推进煤炭行业去产能，坚决完成省政府下达的目标任务。深化“放管服”改革，加快编制公共服务事项清单，推动“双随机、一公开”监管模式全覆盖。创新财政扶持产业发展方式，探索建立财政资金后补助机制。全面实施公务消费网络监管机制改革，强化政府一般性支出约束。加快国有企业、行政事业单位和国有资产管理体制改革，规范国有资产管理，进一步提高运营效率。

　　全面深化农业农村改革。大力推进农业供给侧结构性改革，加速培育农业农村发展新动能。抓好农村土地承包经营权确权登记颁证成果应用，深入推进“三权分置”改革，促进土地资源优化配置。加快建设市县乡三级统一的农村综合产权交易市场。积极开展土地股份合作制和“两权”抵押贷款试点，扎实推进农村集体产权制度改革和农村宅基地改革试点，切实抓好林权改革、水权改革、供销合作社综合改革和农垦改革。

　　稳步实施民生领域改革。突出民生导向，深化教育文化、医药卫生、社会保障、收入分配等社会领域改革。继续深化中小学教师职称制度改革，扎实做好义务教育学校校长教师交流轮岗工作。深入开展公立医院综合改革试点，不断完善公立医院运行机制，加速构建分级诊疗制度体系，着力解决“看病难、看病贵”问题。有序推进户籍制度改革，进一步放宽城镇落户条件，简化户口迁移手续。

　　（五）强化创新驱动，加速发展动能转换

　　创新是引领发展的第一动力。要把科技创新摆在重要位置，深入实施创新驱动战略，真正发挥科技对发展的支撑引领作用。

　　增强协同创新能力。强化企业创新主体地位，促进创新资源要素向企业集聚。鼓励企业加大研发投入，加快科技成果转化，力争全年实施省级重大科技专项1个、申请发明专利800项、新增授权发明专利200项。支持宜春丰城高新技术产业园区创新中国成立家级高新区。加强与国内外知名高校、科研院所的战略合作，联合组建研发平台、技术转移服务中心和科技创新战略联盟。积极推进协同创新，在锂电新能源、中医药等重点产业领域组建3个协同创新体，快速突破一批关键核心技术。

　　激发创新创业活力。放宽政策、放开市场、放活主体，培育形成崇尚创新、鼓励创业、宽容失败的创新创业文化，让创新创业在宜春蔚然成风。积极推广新型孵化模式，鼓励引导科技创新人员、大学生开展创新创业，积极扶持创新型中小微企业做优做强。加快建设众创、众包、众扶、众筹支撑平台，大力发展“创客空间”、“创业咖啡”、“创新工场”等新型众创空间，形成各类创新主体互促、线上与线下互动的生动局面。

　　集聚科技创新要素。创新科技投入、科技管理、科技资源配置和科技评价体系，形成有利于激发创新活力的体制机制。加快科技创新公共服务平台建设，力争全年新增国家级创新平台和载体1个、省级创新平台和载体8个。完善人才评价激励机制和多元化投入机制，让各类人才各得其所、各路高贤大展其长。弘扬“工匠”精神，培育一批支撑发展的实用型、技能型、创新型人才，力争全年新增国家级创新人才和团队1个、省级创新人才和团队4个。

　　（六）坚持绿色发展，打造生态文明“宜春样板”

　　绿色生态是宜春最大的品牌和优势。要强化绿色发展理念，坚守生态底线，做好治山理水、显山露水文章，让青山、碧水、蓝天成为宜春的靓丽名片。

　　深化“三区”发展战略。健全和完善县域经济发展考核激励机制，鼓励各地发挥特色优势，走差异化发展之路，着力打造丰樟高循环区、袁上万绿色区、昌铜四县生态区“三区”发展格局。做强丰樟高循环区，主动对接赣江新区，积极融入南昌大都市区，大力发展循环产业集群，争当全省县域经济排头兵。做大袁上万绿色区，积极构建以低碳工业、生态农业和现代服务业为主要内容的绿色产业体系，培育区域竞争新优势。做美昌铜四县生态区，深入实施“三金”工程，突出培育生态旅游、有机农业、健康养生等特色产业，加快推进省级生态文明先行示范区建设，建成长江中下游生态屏障的“绿心”。

　　加大生态保护力度。坚持保护优先、自然恢复为主，实施山水林田湖生态保护和修复工程。加快生态安全屏障建设，构筑以九岭山自然保护区、官山自然保护区为主体的赣西北—赣西山地森林生态走廊。抓好昌铜、昌栗高速公路沿线补植补造，完成人工造林10万亩、退化林修复13万亩。巩固完善河长组织体系，深入开展“清河行动”，加强畜禽养殖污染防治，保障城乡居民饮水安全。实行最严格的耕地、林地保护制度，加强土地利用规划管控。

　　健全绿色发展机制。推进跨县流域生态保护补偿，实施碳交易和排污权交易试点，构建市场化生态补偿模式。建立健全领导干部任期生态文明责任制度、自然资源资产和环境离任审计以及生态环境损害责任终身追究制。支持奉新、靖安分别开展“生态+”和“生态云”试点。积极开展生态文明创建活动，创建一批绿色机关、绿色学校、绿色社区和绿色家庭。

　　（七）创新社会治理，维护安全稳定局面

　　安全环保是最大的民生。要以最严格的要求落实安全生产责任，以最严厉的手段推进环境治理，以最有效的措施化解矛盾纠纷，切实维护人民群众生命财产安全。

　　全力抓好安全生产。深刻汲取丰城发电厂“11·24”特别重大事故的惨痛教训，坚持警钟长鸣、常抓不懈，进一步做细做实安全生产各项工作。严格落实“党政同责、一岗双责、齐抓共管、失职追责”和“三管三必须”要求，强化安全生产监管，有效遏制安全生产事故发生。紧盯重点行业、重点领域，深入开展安全生产隐患大排查，依法采取“四个一律”强制执法措施，持续保持“打非治违”高压态势。严肃事故查处和责任追究，有效解决失之于软、失之于宽的问题。

　　深入开展环境治理。切实抓好中央环境保护督察组反馈问题整改。加大环境污染隐患排查力度，坚决取缔“十小”企业，及时消除环境事故隐患。大力开展环保执法“零点行动”，严厉打击环境违法行为。全面加强采矿企业、建筑工地、城区道路扬尘污染整治，认真做好老旧机动车及“黄标车”淘汰工作，不断改善环境空气质量。

　　切实维护社会稳定。加强社会治安综合治理智能化建设，坚决落实反恐维稳各项措施，严厉打击“盗抢骗”等违法犯罪行为，奋力争创全国综治“长安杯”。健全完善突发公共事件应急管理机制，扎实做好综合防灾减灾救灾工作。加强重大疫病防治和食品药品安全监管，落实重大决策社会稳定风险评估，提高预防各类风险能力。加强政府债务管理，着力规范各类融资行为，有效防范金融风险。妥善处置历史遗留问题，积极化解信访矛盾纠纷。

　　（八）健全保障体系，促进民生普惠共享

　　改善民生是政府工作的重心所在。要坚持经济发展与改善民生相结合，不断完善改善民生的长效机制，努力让人民群众生活得更加体面、更加幸福、更有尊严。

　　坚决打赢脱贫攻坚战。因地制宜实施精准扶贫，稳步推进十大扶贫工程，努力发展产业、增加就业、促进创业。加大异地搬迁移民力度，有效解决搬迁群众生计问题。加快推进农村低保制度与扶贫开发政策有效衔接，全力做好兜底救助脱贫。争取铜鼓、万载纳入全省财政涉农扶贫资金整合县范围。

　　繁荣发展社会事业。加快乡镇公办中心幼儿园建设、农村义务教育薄弱学校改造和普通高中特色学校创建。创新职业教育人才培养模式，促进职业院校与园区企业对接。实施特殊教育提升计划，鼓励社会力量办学，支持高等院校转型内涵发展。继续开展美丽宜春—全国名家书画明月山活动和宜春颂歌优秀歌曲创作征集活动。加强非物质文化遗产保护传承和合理利用，推进樟树吴城遗址申报世界文化遗产。深入开展“净网”、“清源”等专项行动，全力抓好“扫黄打非”工作。整合城乡居民医保制度，建立公务员医疗补助制度。落实全面两孩政策，提升优生优育服务。积极支持国防和军队调整改革，深入推进军民融合发展，持续强化国防动员和国防教育，扎实做好“双拥”和优抚安置工作。

　　加快建设健康宜春。启动国家健康城市试点工作，提高基层医疗卫生机构服务能力。做好全国基层中医工作先进市复审工作，促进中医药健康服务业发展。推动市人民医院（北院）开业运营，着力打造成为赣西区域医疗中心。全面放开养老服务市场，探索医养结合的养老服务模式。加快新水上运动基地建设，打造一批便民利民的户外健身活动场所。

　　各位代表，本着惠民利民的原则，经过广泛征求意见，今年我们将集中力量为人民群众办好10件看得见、摸得着的民生实事。（1）加快扶贫脱困。完成全市80%以上建档立卡贫困人口脱贫、62个省定贫困村退出任务，力争全年脱贫6.7万人。（2）改善居住条件。新开工城市棚户区改造项目45个、19804套，改造农村危房5388户，确保公共租赁住房分配数达到6563户。（3）提升办学能力。新改扩建学校30所、增加学位4.5万个，其中中心城区新改扩建学校5所、增加学位0.6万个。（4）促进就业创业。免费开展省内工业园区定向培训3.6万人和创业培训1.7万人，发放创业担保贷款13亿元、“财政惠农信贷通”贷款14亿元以上，全年新增城镇就业4.7万人、转移农村劳动力6.7万人。（5）确保饮水安全。重点抓好锦江、袁河（袁州区段）流域环境综合治理，确保水质达标率在90%以上。完成中心城区松江园区域污水截流改造，启动上高备用水源地保丰水库扩建工程及管网建设。（6）实施重大疾病免费救治。完成白内障复明手术2900例、儿童先天性心脏病手术240例、重性精神病救治7900例、尿毒症血透救治1700例、艾滋病机会性感染救治230例，为贫困肢体缺失者安装假肢80条。（7）改善农村生产生活条件。抓好4000个左右村点的新农村建设，新建农村公路（25户以上自然村通水泥路）2500公里，改造农村公路危桥100座。深入实施农村清洁工程，投入资金3亿元，清理垃圾270余万吨。（8）推进文化惠民。完成市美术馆开馆、市群众艺术馆新馆搬迁并免费开放，新改建基层综合文化服务中心700个。免费送戏下乡1600场、农村电影免费放映32166场。（9）健全养老服务体系。扩建市老年大学校舍，打造30个居家养老服务中心示范点，新改扩建敬老院50所，新增养老床位1500张。实施65周岁以上老年人免费乘坐市内公交。（10）方便群众生活。推进市行政服务中心智能化大厅建设。全市新改造城乡农贸市场20个，打通断头路59条，改造背街小巷60条，新改建公厕58个、公共停车位7909个，建设地下综合管廊10.9公里，其中中心城区打通断头路5条，改造背街小巷12条，新改建公厕10个、公共停车位2000个，建设地下综合管廊8.7公里，新购置新能源公交车100辆，加固袁河左岸城防工程2856米。

　　各位代表，要做好全市经济社会发展各项工作，必须进一步加强政府自身建设，努力打造实干型、效能型、服务型、法治型和廉洁型政府。一是强化实干担当。牢固树立发展意识，大力弘扬实干精神，以“功成不必在我”的境界，多做打基础、利长远、增后劲的事情。坚决做到矛盾面前不绕行、困难面前不退缩，敢抓敢管、敢闯敢干，少说多做、说到做到，最大限度汇聚起赶超发展的正能量。二是锤炼过硬作风。深入开展干部作风突出问题集中整治，坚决整肃“慵懒散拖”等问题，以作风大转变助推宜春发展再上新台阶。积极倡导“立说立行、马上就办”的理念，以“踏石留印、抓铁有痕”的劲头，狠抓工作落实。三是提升服务质量。深化政务公开，简化办事环节，增强服务能力，切实提高政府服务水平。恪守为民宗旨，真心实意为群众办好事、解难事，以服务群众的实际成效赢得群众的信任和支持。四是坚持依法行政。牢记权由法定，忠实履行宪法和法律赋予的职责，坚持科学决策、民主决策、依法决策。自觉接受人大法律监督、政协民主监督和社会舆论监督，强化监察、审计等内部监督。五是塑造清廉形象。深化落实全面从严治党“两个责任”，严格遵守“两准则、四条例”，认真执行中央八项规定和国务院“约法三章”，促进政府工作人员廉洁从政、勤勉尽责。严格监管公共资金、公共资源、国有资产，坚决纠正和查处违法违纪行为。

　　各位代表，新的目标鼓舞人心，新的任务催人奋进。我们要牢记使命、肩扛责任，更加紧密地团结在以习近平同志为核心的党中央周围，在省委、省政府和市委的坚强领导下，紧紧依靠全市人民，群策群力、上下一心、左右合力，撸起袖子加油干，为建设美丽宜春决胜全面小康而努力奋斗！
